# Differential Sensing with Replicated Plasmonic Gratings Interrogated in the Optical Switch Configuration

**DOI:** 10.3390/s23031188

**Published:** 2023-01-20

**Authors:** Emilie Laffont, Nicolas Crespo-Monteiro, Arnaud Valour, Pierre Berini, Yves Jourlin

**Affiliations:** 1 School of Electrical Engineering and Computer Science, University of Ottawa, Ottawa, ON K1N 6N5, Canada; 2 Department of Physics, University of Ottawa, Ottawa, ON K1N 6N5, Canada; 3 Laboratoire Hubert Curien, Université de Lyon, UMR CNRS 5516, 42000 Saint-Etienne, France; 4 Nexus for Quantum Technologies Institute, Advanced Research Complex, 25 Templeton Street, Ottawa, ON K1N 6N5, Canada

**Keywords:** surface plasmon resonance, diffraction grating, optical switch, bulk sensing, optical sensors

## Abstract

A new plasmonic configuration is proposed for application in a sensor and demonstrated for the detection of variations in the bulk refractive index of solutions. The configuration consists of monitoring two diffracted orders resulting from the interaction of a TM-polarized optical beam incident on a grating coupler, operating based on an effect termed the “optical switch”. The two monitored diffracted orders enable differential measurements which cancel the drift and perturbations common to both, leading to an improved detection limit, as demonstrated experimentally. The measured switch pattern associated with the grating coupler is in good agreement with theory. Bulk sensing is demonstrated under intensity interrogation via the sequential injection of solutions comprised of glycerol in water into a fluidic cell. A limit of detection of about $10^{-6}$ RIU was achieved. The optical switch configuration is easy to implement and is cost-effective, yielding a highly promising approach for the sensing and the real-time detection of biological species.

## 1. Introduction

Biosensors and their performance characteristics have been the subject of vigorous global research for several decades. However, the COVID-19 pandemic highlighted and increased the need for portable and cost-effective biosensors to address challenges in large-scale testing and screening. Surface plasmon resonance (SPR) biosensors [1,2] and their integration into other systems [3,4] have been identified as a highly promising approach, offering the benefits of label-free, real-time, rapid detection to overcome the limitations of the laboratory-based techniques in current use, such as ELISA (enzyme-linked immunosorbent assay) [5] and RT-PCR (reverse transcription polymerase chain reaction) [6]. The most widespread SPR detection configuration is based on a prism coupler [7], and is rather cumbersome and expensive [8], attributes which do not align well with point-of-care (POC) settings.

Portable and miniaturized surface plasmon (SP) sensors have been investigated and developed using, for example, configurations based on gratings [9,10] and waveguide couplers [11,12,13]. Such configurations are easy and cost-effective to implement and integrate with microfluidics on a chip and with external fluid components. Even though waveguide configurations certainly benefit from the highest degree of miniaturization, they are less tunable than the grating coupler, where the shape of the profile [14], the periodicity, and the depth are all parameters that have an influence on the response of the system [15,16]. Furthermore, sensors based on Blu-ray nanoslits [17], silver nanotriangle arrays [18], nanoantennas [19], and nanoholes [20] have been developed, yielding a performance level sufficient for the detection of diseases [21,22].

Recently, a new surface plasmon detection configuration termed the “optical switch” [23,24,25] was proposed and justified theoretically [26] as a promising method of detection based on a grating coupler. This approach involves monitoring two diffracted orders, the −1st and the 0th diffracted orders, produced by the interaction of an incident light beam with a deep metal grating. The intensity of these orders vary contrastingly as the refractive index (RI) varies near the interface of the grating with the sensing medium. Therefore, monitoring two orders instead of only one allows the cancellation of noise and fluctuations due to common perturbations by adopting a differential measurement scheme as the real-time difference in the intensities of both diffracted orders. This configuration also considerably simplifies interrogation of the sensor, with only one optical source and two detectors aligned at fixed angles, measuring the intensity of both diffracted orders instead of a spectral or angular measurement, thereby reducing the sensor size and cost.

In this paper, we propose a new, cost-effective, easy-to-implement plasmonic sensor based on an optical switch configuration, offering high detection performance, good miniaturization potential, and the ability to cancel common noise by a differential measurement. Our approach of producing a grating master then applying a replication process to produce the sensors is aligned with low-cost manufacturing.

## 2. Materials and Methods

### 2.1. Materials

Acetone HPLC grade ≥99.9%, 2-isopropanol (IPA), and glycerol were obtained from Sigma-Aldrich (Oakville, ON, Canada). AMOPRIME and AMONIL MMS1 were provided by AMO. Photoresist Shipley S1828 and developer MF319 were bought from MICROPOSIT. The EPDM (ethylene propylene diene monomer) O-ring used to delimit the fluidic cell was obtained from PAUL and is identified by the product number NG0201023095.

### 2.2. Production of Grating Masters

**Figure 1:**
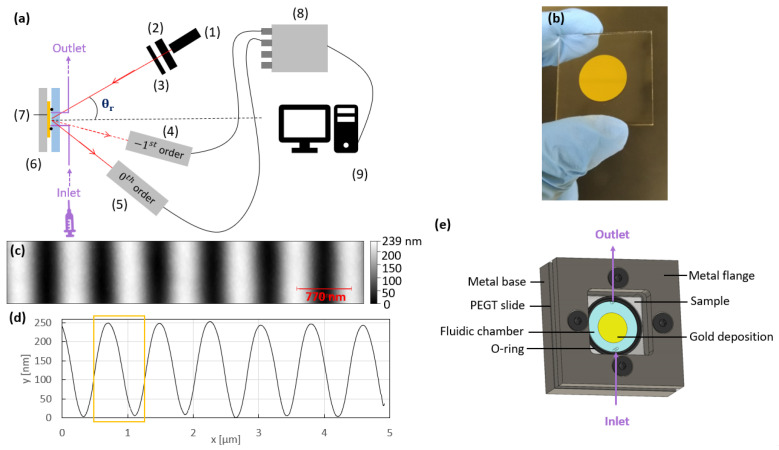
(**a**) Setup used to perform the measurements, comprising an 850 nm wavelength laser diode (1), a polarizer (2), an aperture (3), and two photodiodes (4) and (5) to measure the power in the −1st (dashed red line) and the 0th (solid red line) orders diffracted from the gold-coated grating (6) placed within a fluidic cell (7) into which fluids are injected via peek tubing interfaces (purple) connected to a syringe pump. A DAQ (data acquisition) device (8) and a computer (9) were used to record the measurements. (**b**) Picture of the gold-coated grating sample on a 26 × 26 mm${}^{2}$ glass slide. The sample consists of a sinusoidal grating replicated in amonil covered by a thin chromium layer and a 121 nm-thick gold layer. (**c**) Partial 2D AFM scan of the corrugated gold-coated grating replica used to perform the measurements. (**d**) AFM profile of the corrugated gold-coated grating replica used to perform the measurements. (**e**) Schematic of the sensor (item (6) in Part (**a**)) showing the fluidic inlet and outlet.

The grating masters were produced by laser interference lithography (LIL) [27]. First, a 26 × 26 mm${}^{2}$ glass substrate was cleaned by three sequential steps: ultrasonic cleaning in acetone for 10 min, ultrasonic cleaning in ethanol for 10 min, and static incubation in de-ionized water for 10 min. After drying under nitrogen gas, a thin positive photoresist (Shipley S1828) layer was deposited on the cleaned substrates by spin coating, then soft-baked for 1 min at 60 ${}^{\circ }$C to evaporate the solvent and increase the density of the layer. Then, the samples were exposed to a uniform He-Cd laser beam at a wavelength of λ = 442 nm and an incident power of 204 µW/cm${}^{2}$ for 115 s to reach the linear operating regime of the photoresist. A second exposure was then applied, consisting of the superposition of two balanced and coherent beams from the same laser at an incident power of 408 µW/cm${}^{2}$ for 115 s, to produce the desired interferometric sinusoidal profile. During this second exposure, the grating period in the photoresist was fixed at 770 nm by the laser wavelength and the angle of incidence of the beams (controlled by a Labview program), giving rise to a fringe pattern where the two beams overlap. This period was chosen based on simulations with the software MC Grating [28] by considering two criteria: (i) maximizing the lateral and central extrema of the optical switch pattern to gain sensitivity and dynamic range (as discussed further below), (ii) while maintaining a high enough angular spread between working points to easily reach each of them without obstructing the incoming beam with the photodiodes (as shown in Figure 1a). Indeed, with a large period, the working points become too close to each other to be distinguished or even reached with the setup illustrated in Figure 1a. However, a small period results in smaller transient variations around each working point and also in lower performance (as shown in Figure A2a). In the same way, a non-optimized depth reduces the amplitude of the central extrema and thus the amplitude of the transient variations (as shown in Figure A2b). Next, the samples were developed in MF319 for 9 s to remove the areas which became soluble after the second exposure. Then, the samples were flushed with de-ionized water and dried under nitrogen gas. For the samples used in this paper, a grating depth of 285 nm was desired and achieved as verified by AFM characterization. The period of these samples was deduced from a measurement of the Littrow angle [29].

### 2.3. Production of Grating Replicas

LIL is a fast and cheap method to produce large-area periodic nanostructures. However, the profile of the photoresist produced by this technique becomes modified and damaged after a prolonged exposure to fluids encountered in biosensing, which compromises the reliability and the repeatability of the measurements. Thus, the master gratings produced in photoresist by LIL were replicated using a nanoimprinting process adapted from [30]. Firstly, a PDMS (polydimethylsiloxane) stamp fabricated from a master grating was applied to a thin Amonil MMS1 layer used as the imprint resist. The latter was deposited by spin-coating on a clean 26 × 26 mm${}^{2}$ glass substrate after previous deposition of Amoprime as adhesion promoter. Secondly, a low imprint pressure was applied using a printing press to the PDMS stamp in contact with the sample. Thirdly, the sample was illuminated by a UV lamp (Ucube 365-100-2) provided by Uwave to harden the imprint resist. Finally, the stamp was released from the replica, which was then soft-cured at 60 ${}^{\circ }$C for 1 min.

### 2.4. Deposition of Thin Metal Layers

To form the final grating couplers, chromium and gold layers were deposited sequentially on the amonil replicas, as shown in Figure 1b, by thermal evaporation. A thin chromium layer was used as an adhesion promoter for the gold layer. The deposition rates and the final thicknesses were 3.3 Å/s and 7.7 nm for the chromium film, respectively, and 13.4 Å/s and 120.7 nm for the gold layer, respectively. These two layers were deposited with a vacuum chamber pumped to a pressure of about $10^{-6}$ mBar. The thickness of the gold layer was chosen to be greater than 100 nm to prevent transmitted orders from emerging during the detection measurements. For the sample used here, an average depth of 228 nm was measured by AFM characterization, as shown Figure 1c,d.

### 2.5. Sensing Platform

To perform the measurements, a grating was placed in a custom fluidic cell, as illustrated in Figure 1e. The cell was closed by a PETG (polyethylene terephthalate glycol) lid with a machined trench housing a 20.35 mm diameter, 1.78 mm-thick O-ring to seal and delimit the sensing area. The lid was drilled with two holes through which peek tubing was threaded and glued (Krazy Glue${}^{\mathrm{TM}}$ from Canadian Tire, Ottawa, ON, Canada), enabling solutions to be injected using a syringe pump. A metal base was used to support the grating, and a flat square metal flange was used to secure the PEGT window to the base by four screws, ensuring that a uniform pressure was applied by the O-ring to the substrate. The grating area was designed to be smaller than the diameter of the O-ring to ensure there was no contact between the O-ring and the sensing surface.

### 2.6. Interrogation Setup

The sensor sketched in Figure 1e and described in Section 2.5 was integrated into the setup sketched in Figure 1a, with which the measurements were obtained. A collimated beam from a laser diode emitting at the free-space wavelength of $\lambda_{0}$ = 850 nm probed the grating, which was placed inside the flow cell, and solutions were injected via the peek tubing connected to a syringe. An IR-polarizer was used to fix the incident polarization to TM (transverse magnetic) and an aperture removed the background light. The angle of incidence was set using a rotation stage holding the cell and grating aligned along the central rotation axis using an xy stage. Two 5 × 5 mm${}^{2}$ Si-based photodiodes were fixed to tracks connected to two other rotation stages with their axes of rotation aligned with those of the rotational stage controlling the cell orientation. Both photodiodes were used to measure the power diffracted by the 0th and the −1st orders. Photodiode current was converted to voltage using a transimpedance circuit giving an output signal (voltage) proportional to the incident optical signal. Labview software was used to perform data acquisition from both photodiodes.

### 2.7. Solution Preparation

To demonstrate the optical switch configuration for the detection of small RI variations, three solutions ($s_{1}$, $s_{2}$, and $s_{3}$) comprised of a mixture of de-ionized water and glycerol were prepared to produce refractive index increments of about $10^{-3}$. De-ionized water was used as the reference solution, $s_{0}$, to establish the baseline of the sensorgrams presented in Section 3. The RI of the solutions $s_{0}$, $s_{1}$, $s_{2}$, and $s_{3}$ were 1.3211, 1.3265, 1.3283, and 1.3311, respectively, as measured using an instrument based on a prism coupler (Metricon, Model 2010, Prism 200-P1) at $\lambda_{0}$ = 1312 nm.

## 3. Results and Discussion

### 3.1. Theoretical and Experimental Switch Patterns

The grating optical switch transfers energy between two orders diffracted by a deep sinusoidal metallic grating, as the angle of incidence is varied over a few degrees about a working point (defined further below). The switching operation requires that the incident beam is simultaneously coupled to two SP modes, one propagating co-directionally and the other contra-directionally from the incident beam.

**Figure 2:**
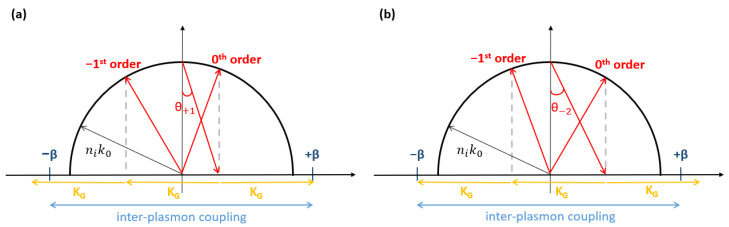
(**a**) Co-directional and (**b**) contra-directional coupling schemes, illustrating diffraction and SP interaction processes. $\theta_{+1}$ and $\theta_{-2}$ are the angles of the incident beam. $K_{G}$ stands for the grating wavenumber (momentum). +β and −β denote the propagation constants associated with the forward and the backward SPs, respectively. $n_{i}$ and $k_{0}$ represent the RI of the sensing medium and the wavenumber of the incident beam, respectively.

The Ewald circles sketched in Figure 2 illustrate two angles of incidence, $\theta_{+1}$ and $\theta_{-2}$, along with diffraction into different channels. At the angle of incidence $\theta_{+1}$, the incident beam is coupled to the SP mode via the +1st order of the grating which provides $+K_{G}$ of momentum (Figure 2a). The SP mode thereby excited is co-propagating, and has a wavenumber +β (propagation in the forward direction). This excitation scheme is termed co-directional coupling. At the angle of incidence $\theta_{-2}$, the incident beam is coupled to the SP mode via the −2nd order of the grating, which provides a momentum of $-2K_{G}$ (Figure 2b). The SP mode thus excited is counter-propagating, with a wavenumber of −β (propagation in the backward direction), and this excitation scheme is termed contra-directional coupling. In both cases, the co-propagating and the contra-propagating SPs are simultaneously coupled via the ±3rd order of the grating, which provides a momentum of $\pm 3K_{G}$ and approximately satisfies the momentum conservation condition.

**Figure 3:**
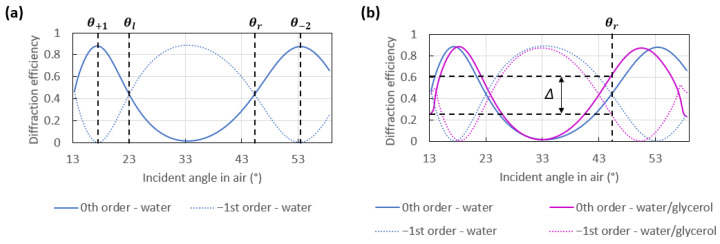
(**a**) Computed angular response of an ideal sinusoidal grating of period 770 nm and depth 228 nm, formed as a 100 nm-thick gold layer, covered by water (*n* = 1.33), and under plane wave incidence at $\lambda_{0}$ = 850 nm. The diffraction efficiencies of the 0th and −1st orders produce a switching pattern as the angle of incidence is varied. (**b**) Computed angular response for a cover solution comprised of water and glycerol (*n* = 1.35, pink curves) for the same grating in part (**a**). The difference in the diffraction efficiencies of the 0th and −1st orders, Δ, at $\theta_{r}$ is no longer zero (as it was for water, *n* = 1.33, blue curves).

The computed angular response of the optical switch is given in Figure 3a, where the two working points, denoted $\theta_{l}$ and $\theta_{r}$, are defined as the angles at which the diffraction efficiencies of the −1st and the 0th orders are equal. These responses were computed using the software MC Grating based on Chandezon’s method [31], by modelling one period of the ideal grating bounded by water.

**Figure 4:**
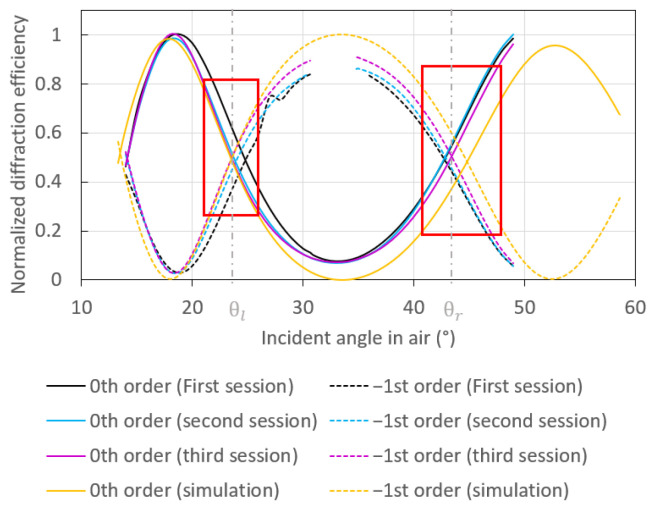
Normalized measured switch patterns (black, blue, and purple) obtained at different areas on the grating. $\theta_{l}$ and $\theta_{r}$ correspond to the left and right working points, respectively, where the diffraction efficiencies associated with the 0th and −1st orders are equal. Both red boxes delimit the linear region of each working point. The normalized theoretical switch pattern (yellow) was computed using MC Grating. The period used in the computations was extracted from an AFM scan of the plasmonic grating tested, as highlighted by the yellow box in Figure 1d.

Experimental switch patterns were obtained using the set-up sketched in Figure 1a for water injected into the flow cell. The experimental switch patterns presented in Figure 4 as the black, blue, and purple curves were normalized by dividing the respective measurements (photodiode output voltage of the transimpedance circuit) by the maximum value achieved for each. The normalized experimental switch patterns plotted in Figure 4 were measured at three different areas on the sample. They are quite similar, demonstrating that the sample is homogeneous in its fabrication.

**Figure A1:**
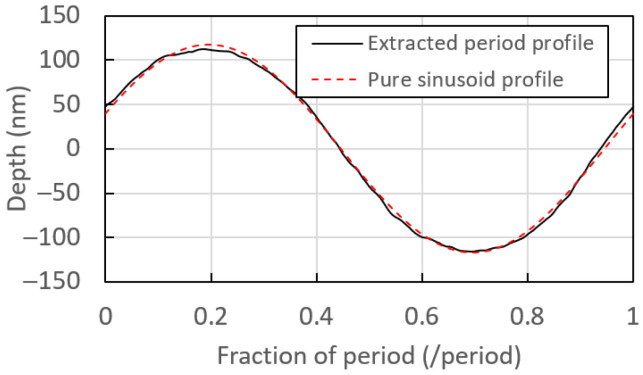
Profile of one period (solid black curve) extracted from the AFM scan within the yellow box in Figure 1d, compared with a pure sinusoid (dashed red curve).

**Figure A2:**
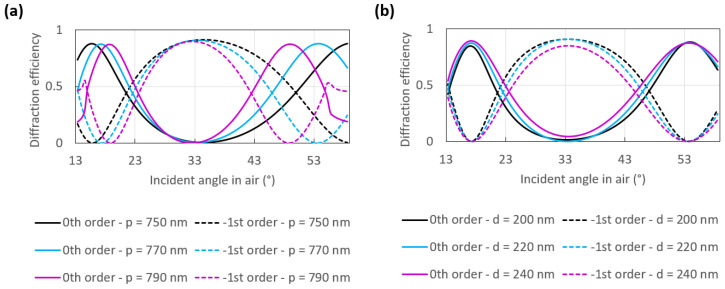
(**a**) Computed angular response for an ideal sinusoidal grating of depth 220 nm, covered by water (*n* = 1.33), under plane wave incidence at $\lambda_{0}$ = 850 nm depending on the period. (**b**) Computed angular response for an ideal sinusoidal grating of period 770 nm, covered by water (*n* = 1.33), under plane wave incidence at $\lambda_{0}$ = 850 nm depending on the depth. The switch patterns were computed using MC Grating.

The theoretical response, plotted as the yellow curves, was also obtained with the software MC Grating, by modelling one period of the fabricated grating. The modelled period is highlighted by the yellow box in Figure 1d, extracted from an AFM scan of the tested grating. The measured period was compared with a pure sinusoidal profile in Figure A1, revealing a good fidelity of the fabricated structure. The theoretical response was normalized in the same way as the experimental response (using the computed diffracted waves), so they could be directly compared.

The slight mismatch between the three measured switch responses and the computed response can be explained in several ways. First, the software used to obtain the theoretical switch pattern assumes a perfectly uniform grating, infinitely periodic of the period extracted from the AFM profile. However, even though the grating is rather homogeneous, its period varies slightly over its length, as observed in Figure 1d. Furthermore, the model does not take into account the interaction between the incident beam and the PEGT lid of the fluidic cell sketched in Figure 1e. Indeed, the model assumes that the beam is incident from water, but as previously described, the incoming beam emerges from a laser diode and propagates in air before interacting with the PEGT lid, then emerges in water to excite the grating. This interaction results in multiple reflections at the air/PEGT and PEGT/water interfaces, causing power loss. Additionally, roughness [32] and potential discrepancies between the permittivity of gold used in the software and that associated with the sample could partially explain the difference between theory and experiment.

SPs are sensitive to the cover medium bounding the metal surface; therefore, a change in the refractive index of this medium induces a change in the optical switch pattern, as shown in Figure 3b. Specifically, if the angle of incidence is fixed to $\theta_{l}$ or $\theta_{r}$ for a reference cover medium such as water, then the diffraction efficiencies of the −1st and the 0th orders will change (and no longer be equal) if the refractive index of the cover medium changes. Thus, the difference in the powers emerging from these orders can be used to assess refractive index variations along the metal surface or to detect the immobilization of targeted biomolecules by a biorecognition layer on the metal surface.

### 3.2. Sensitivity

To demonstrate the optical switch as a piece of equipment to detect small RI variations, four solutions of different RI were sequentially injected into the fluidic cell. These solutions were mixtures with different proportions of water and glycerol, with a RI increment of 2 ×$10^{-3}$ refractive index unit (RIU), as verified independently by measurement with a Metricon prism coupler. Two cycles of solution injection were carried out to demonstrate the repeatability of the measurements under a continuous flow-rate of 160 µL/min. The angle of incidence was initially fixed to the left working point, $\theta_{l}$, then to the right working point, $\theta_{r}$, following Figure 4.

**Figure 5:**
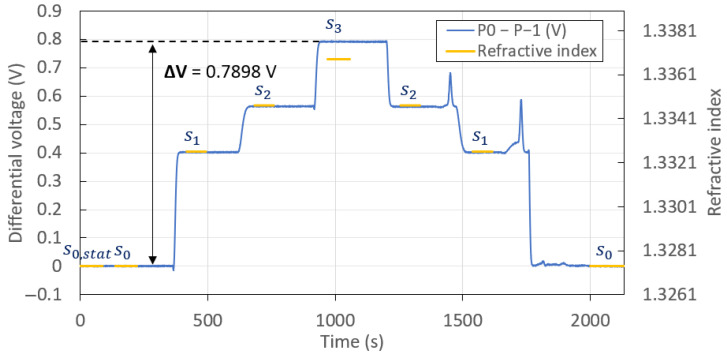
Response of the plasmonic grating with the profile illustrated in Figure A2a, to the sequential injection of solutions (glycerol in water) of RI summarized in Table 1 at the angle of incidence $\theta_{l}$ (left working point) and a continuous flow rate of 160 µL/min. The injection of all solutions except for $s_{3}$ was cycled once to demonstrate repeatability. The step associated with solution $s_{0,stat}$ corresponds to the solution $s_{0}$ under static conditions (syringe pump off). The steps associated with the solutions of different RI correspond to the time over which the solutions were injected. The RI calculated at $\lambda_{0}$ = 850 nm from those measured at $\lambda_{0}$ = 1312 nm (reported in Table 1) is shown as the yellow horizontal lines for reference.

The power carried by the 0th and −1st orders should be equal when the angle of incidence is fixed to one of the working points and the reference solution ($s_{0}$) is injected. Figure 5 confirms this, as the measured baseline signal, taken as the differential output voltage associated with the injection of solution $s_{0}$ (0 to 400 s), is equal to 0 V for operation at the left working point, $\theta_{l}$. (The portion associated with solution $s_{0,stat}$ corresponds to the solution $s_{0}$ under static conditions, i.e., syringe pump off.) A very strong correlation is observed between the differential signal and the RI of the injected solutions. Moreover, the same output powers and differential signals were recovered each time the same solution was injected, which demonstrates the repeatability of the measurements. The slight mismatch between the RI and the differential signal measured for solution $s_{3}$ is likely due to an error in RI measurement of this solution with the Metricon, because the agreement between the RI and the differential signal is excellent for the other solutions.

Table 1 summarises the mean differential voltage measured for each injected solution, along with the system noise (taken as the standard deviation over time of the differential voltage), for the measurements given in Figure 5. As summarized in Table 1, the noise for each injection of solution was rather steady at about 0.1 mV, except for the last two injections due to mild fluidic disturbances. The larger transient variations in differential output signal observed after the second injection of solutions $s_{2}$ and $s_{1}$ may be due to slower fluid exchanges. The slight instability in the measured signal between 1817s and 1999s observed in Figure 5 (during the second injection of $s_{0}$) is due to a mild disturbance in the fluidic system, likely caused by repeatedly turning on and off the syringe pump, and is not representative of the system noise. Thus, the noise given for the second injection of solution $s_{0}$ in Table 1 was assessed between 2000s and 2205s.

The angle of incidence was then adjusted to $\theta_{r}$ (right working point), while maintaining incidence on the same region of the grating, and the same solutions were injected in the same order. The sensorgram thus obtained (not shown) was very similar in appearance to that plotted in Figure 5. Table 2 summarises the mean differential voltage measured and the system noise for each injected solution obtained in this case.

**Figure 6:**
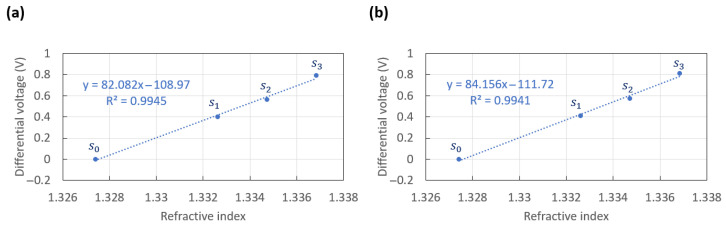
Measured differential voltage vs. refractive index ($\lambda_{0}$ = 850 nm) of the solutions injected, taken as the mean value measured for the first injection of each solution reported in (**a**) Table 1 at $\theta_{l}$, and (**b**) Table 2 at $\theta_{r}$. The dotted lines correspond to the best fitting linear models.

The differential voltage should vary linearly about each working point with the RI of the solutions ($\lambda_{0}$ = 850 nm). Figure 6 plots the measured differential voltage vs. the refractive index of the solutions injected. The data points were taken as the mean value measured for the first injection of each solution, as summarised in Table 1, for operation at the left working point ($\theta_{l}$), and Table 2 for operation at the right working point ($\theta_{r}$). The dotted lines plotted on each graph correspond to the best fitting linear models (equations and $R^{2}$ goodness of fit given in inset).

The average of the standard deviations, $\delta_{a}$, associated with the differential measurements of Figure 5, taken over all solution injections summarized in Table 1, was $\delta_{a}=1.88\times 10^{-1}$ mV. The largest change in differential output was 0.7898 V, achieved from the first cycle of injection, $s_{0}$ to $s_{3}$ (Figure 5), for which $n_{s_{0}}$ = 1.3274 and $n_{s_{3}}$ = 1.3368. These results imply a signal-to-noise ratio (SNR) of $\Delta V\delta_{a}$ = 4191 for the left working point. Assuming that the minimum SNR required for reliable detection would be equal to two, a limit of detection (LOD) of $4.48\times 10^{-6}$ RIU was achieved for the left working point, as defined by:

(1) $$LOD=2\cdot \frac{\delta_{a}}{\Delta V}\cdot \left(n_{s_{3}}-n_{s_{0}}\right)$$

The average standard deviation, $\delta_{a}$, associated with the differential measurements reported in Table 2 was $\delta_{a}=3.18\times 10^{-1}$ mV. The largest variation in signal of 0.8102 V was achieved between the first injections of solutions, from $s_{0}$ to $s_{3}$, with $n_{s0}$ = 1.3274 and $n_{s3}$ = 1.3368. These results imply an SNR of 2550 for the right working point and an LOD of $7.37\times 10^{-6}$ RIU (Equation (1)). This performance was slightly worse than that obtained for the left working point, mostly due to the standard deviations being higher for the measurements at $\theta_{r}$. However, the LOD achieved for both working points was similar, and sufficiently competitive for disease detection in complex fluids [34,35].

### 3.3. Noise and Differential Measurement

**Figure 7:**
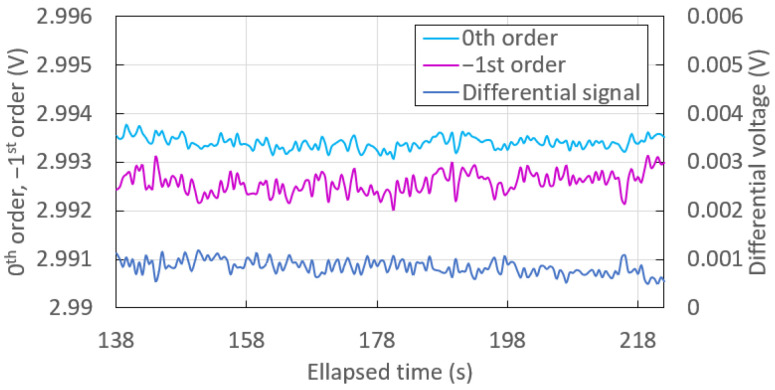
Time traces associated with the first injection of solution, $s_{0}$, with natural noise.

An advantage of the optical switch configuration lies in its ability to perform differential measurements, thus cancelling noise due to common fluctuations. Figure 7 shows the time traces of each monitored order and of the differential signal recorded during the first injection of solution, $s_{0}$, on the sensorgram plotted in Figure 5. These traces reveal the baseline noise constituents of the system.

In this case, the noise of the differential signal was not significantly reduced compared to that associated with each monitored order. Indeed, the standard deviation associated with the 0th order, reported in Table 3, was even slightly lower than that of the differential signal because of the 2-fold higher standard deviation of the −1st order. In this case, very few common fluctuations are recorded and cancelled from the measurements.

**Figure 8:**
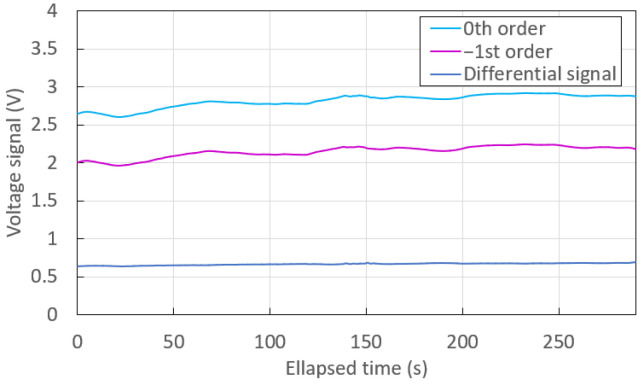
Time traces associated with the injection of a solution (RI of 1.3295 at $\lambda_{0}$ = 1312 nm) with artificial noise introduced by manipulating the syringe.

However, if we (artificially) introduce common noise into the system, the interest in monitoring two orders and in differential detection becomes clear. Indeed, Figure 8 shows the time traces of the monitored orders and of the differential signal measured during the injection of a solution, with disturbances introduced by manipulating the syringe. Contrary to Figure 7, one can see that the dark blue curve associated with the differential signal remains steady, whereas the light blue and pink curves of the 0th and the −1st orders are noisy and unstable, drifting with time. These observations are supported by the data reported in Table 4, from which we note that the standard deviation of the monitored orders were approximately 8-fold higher than that of the differential signal. Therefore, the differential signal does not exhibit any drift, and has lower noise compared with the signal of the individual orders.

## 4. Conclusions

A new plasmonic sensor configuration based on the optical switch effect was successfully implemented and demonstrated by detecting variations in the bulk refractive index of solutions. The measured switch patterns of gold-coated replicated gratings agree very well with theory. The grating-based devices have the ability to cancel noise and drift due to common fluctuations by monitoring two complementary diffracted orders and taking their difference. An LOD in the range of $10^{-6}$ RIU was achieved at each working point of the switch pattern. Improvements to the design of the fluidic cell to reduce its volume, and consequently the flow-rate at which the solutions are injected, would ease the pressure required for timely fluidic exchanges and reduce the noise in the system. The high sensitivity and low cost of producing replica gratings make a compelling case for various biosensing applications in a POC setting.

## Figures and Tables

**Table 1:** Refractive index (RI) at $\lambda_{0}$ = 1312 nm and $\lambda_{0}$ = 850 nm, mean (time-averaged) differential output voltage, and standard deviation over time of the differential output voltage for each solution injected, as measured at the angle of incidence $\theta_{l}$ (left working point, Figure 5). The RI at $\lambda_{0}$ = 850 nm were calculated using the dispersion coefficients of water and glycerol and the Sellmeier dispersion equation reported in [33].

Solution	RI ($\lambda_{0}$ = 1312 nm)	RI ($\lambda_{0}$ = 850 nm)	Mean (V)	Standard Deviation (mV)
$s_{0,stat}$	1.3211	1.3274	0.00062	0.18
$s_{0}$	1.3211	1.3274	0.00083	0.15
$s_{1}$	1.3265	1.3326	0.40162	0.14
$s_{2}$	1.3283	1.3347	0.56299	0.11
$s_{3}$	1.3311	1.3368	0.79070	0.14
$s_{2}$	1.3283	1.3347	0.56193	0.14
$s_{1}$	1.3265	1.3326	0.40099	0.24
$s_{0}$	1.3211	1.3274	0.00037	0.39

**Table 2:** Refractive index (RI) at $\lambda_{0}$ = 1312 nm and $\lambda_{0}$ = 850 nm, mean (time-averaged) differential output voltage, and standard deviation over time of the differential output voltage for each solution injected, as measured at the angle of incidence $\theta_{r}$ (right working point). The RI at $\lambda_{0}$ = 850 nm were calculated using the dispersion coefficients of water and glycerol and the Sellmeier dispersion equation reported in [33].

Solution	RI ($\lambda_{0}$ = 1312 nm)	RI ($\lambda_{0}$ = 850 nm)	Mean (V)	Standard Deviation (mV)
$s_{0,stat}$	1.3211	1.3274	0.00059	0.30
$s_{0}$	1.3211	1.3274	−0.00025	0.27
$s_{1}$	1.3265	1.3326	0.40876	0.25
$s_{2}$	1.3283	1.3347	0.57487	0.28
$s_{3}$	1.3311	1.3368	0.81001	0.26
$s_{2}$	1.3283	1.3347	0.57303	0.26
$s_{1}$	1.3265	1.3326	0.40529	0.31
$s_{0}$	1.3211	1.3274	−0.00245	0.60

**Table 3:** Mean (time-averaged) differential output voltage and standard deviation over time of the differential output voltage associated with the injection of solution, $s_{0}$, shown in Figure 7 between 138 and 222 s.

Signal	Mean (V)	Standard Deviation (mV)
0th order	2.99340	0.13
−1st order	2.99257	0.22
Differential	0.00083	0.15

**Table 4:** Mean (time-averaged) differential output voltage and standard deviation over time of the differential output voltage associated with the injection of a solution (RI of 1.3295 at $\lambda_{0}$ = 1312 nm) as shown in Figure 8.

Signal	Mean (V)	Standard Deviation (mV)
0th order	2.81716	88.09
−1st order	2.14836	75.60
Differential	0.66880	13.86

## Data Availability

All data underlying the results of the paper are present in the paper.
